# A hybrid simulation-machine learning proxy model for waterflood design optimization in the Bahariya Formation

**DOI:** 10.1038/s41598-026-49561-5

**Published:** 2026-05-01

**Authors:** Ramy Gad, Adel M. Salem, Omar M. El Farouk, A. N. El-Hoshoudy

**Affiliations:** 1 Department of Petroleum Engineering, Petrosannan Petroleum Company, Cairo, Egypt; 2 Department of Petroleum Engineering, Faculty of Engineering and Technology, Future University in Egypt (FUE), Cairo, 11835 Egypt; 3 Department of Petroleum Engineering, Faculty of Petroleum and Mining Engineering, Suez University, Suez, 11252 Egypt; 4 Faculty of Applied Engineering, University of Antwerp, 2000 Antwerp, Belgium; 5 PVT Lab, Production Department, Egyptian Petroleum Research Institute, Cairo, 11727 Egypt; 6 PVT Service Center, Egyptian Petroleum Research Institute, Cairo, 11727 Egypt

**Keywords:** Waterflooding optimization, Machine learning, Recovery factor prediction, Hybrid modeling, Reservoir management, Energy science and technology, Engineering, Environmental sciences, Hydrology, Mathematics and computing, Solid Earth sciences

## Abstract

This study presents a novel hybrid methodology combining machine learning (ML) with conventional reservoir simulation to optimize waterflooding in the geologically complex Bahariya Formation, Western Desert, Egypt. The research addresses the critical need for accurate oil recovery efficiency (RF%) prediction by developing and deploying robust data-driven models. Linear regression quantified the impact of nine key parameters across three injection patterns. A pivotal finding from numerical simulation was the ranking of Ultimate Oil Recovery (UOR) for each pattern under identical conditions: Peripheral flooding achieved the highest recovery at 44.7%, followed by Staggered Line Drive (SLD) at 39.4%, and the 5-Spot pattern at 33.7%. Our ML models demonstrated exceptional predictive accuracy, with R² scores of 0.974, 0.972, and 0.953 for the respective patterns, and a correspondingly low RMSE range of 0.0057–0.0085. Permutation importance analysis quantified the dominant influence of residual oil saturation (Sor), accounting for 38–42% of predictive power. Crucially, the models revealed distinct, pattern-dependent control parameters: injection rate (WINJ) showed markedly higher sensitivity in the Peripheral pattern (23% contribution), while API gravity was the second most important feature for the 5-Spot pattern (18% contribution). The findings provide a validated, efficient framework for rapid waterflooding scenario screening and optimization. This work highlights the substantial potential of hybrid AI-numerical approaches to enhance decision making and challenges conventional assumptions about pattern selection, demonstrating that the optimal pattern is profoundly dependent on specific reservoir characteristics. Field engineers can apply these insights to optimize injection strategies during reservoir development planning, potentially increasing recovery factors while reducing reliance on time-intensive simulation runs.

## Introduction

Waterflooding is one of the most widely applied secondary oil recovery methods used to enhance hydrocarbon production from depleted reservoirs^1^. It involves the injection of water into the reservoir to maintain pressure and displace hydrocarbons toward production wells, thereby increasing the overall recovery factor (RF) of oil from the reservoir^2^. Since its inception in the 1950 s, waterflooding has undergone significant technological evolution, transitioning from simple conceptual models to highly sophisticated designs that integrate reservoir heterogeneity, advanced fluid characterization, and numerical simulation^3,4^. In the early stages, waterflood design relied on simplified, two-dimensional models with limited consideration of complex reservoir heterogeneities and multiphase flow dynamics^5,6^. The introduction of numerical reservoir simulation in the 1960–1970 s marked a transformative step, enabling engineers to simulate reservoir behavior under varying injection and production scenarios^4^. Early simulators, however, were constrained by computational capacity, limiting the fidelity and scale of predictive models^7,8^.

By the 1980 s, advancements in computing and reservoir characterization enabled the integration of three-dimensional (3D) simulation, providing a more accurate representation of reservoir geometry and flow dynamics^4,9^. At the same time, enhanced oil recovery (EOR) techniques, such as polymer flooding, surfactant flooding, and CO₂ injection, were introduced to complement waterflooding, necessitating a more rigorous approach to operational design and monitoring^10,11^. The 1990 s and 2000 s marked a paradigm shift with the emergence of integrated reservoir management workflows. These workflows combined multidisciplinary expertise from reservoir engineering, geology, geophysics, and production engineering, improving waterflood design reliability and economic feasibility^12^. The growing availability of large datasets further facilitated the integration of machine learning (ML) and artificial intelligence (AI) techniques into reservoir simulation and optimization processes^13,14^.

Recent studies have demonstrated the power of AI-driven approaches for EOR optimization^15^. An integrated experimental design with four machine learning algorithms, Quadratic Equation, Fuzzy Logic Genetic Algorithm, Multivariate Additive Regression Splines (MARS), and Generalized Boosted Modeling (GBM) to construct reduced physics proxy models for CO₂-EOR in the Rumaila oil field’s clastic reservoir. Their GBM algorithm achieved exceptional accuracy with an adjusted R² of 0.9973, demonstrating that advanced machine learning can effectively replace complex compositional simulations while maintaining prediction accuracy. Concurrently, combined Particle Swarm Optimization (PSO) with polynomial and nonparametric regressions to optimize cyclic CO₂ flooding in shale oil reservoirs, achieving an incremental oil production increase of 322,675 surface barrels through optimization of eight operational parameters, including injection, soaking, and production durations. These studies underscore the transformative potential of hybrid AI-numerical approaches for rapid EOR process evaluation and optimization^16^.

Modern waterflooding design now leverages hybrid workflows that couple physics-based numerical simulators with data-driven ML algorithms. These approaches enhance predictive accuracy, optimize field development strategies, and reduce operational risks^17,18^. Such integration has allowed oil and gas operators to transition toward sustainable and cost-effective recovery solutions, incorporating strategies like water management, carbon capture and storage (CCS), and renewable energy utilization in field operations^19,20^.

The Bahariya Formation in Egypt’s Western Desert exemplifies the type of complex reservoir that urgently requires such integrated approaches. Characterized by significant lithological heterogeneity resulting from its fluvial deltaic depositional environment, the formation exhibits pronounced permeability contrasts, discontinuous sand bodies, and complex fluid flow pathways that challenge conventional simulation methods. Furthermore, limited well control, sparse core data, and incomplete production histories create substantial uncertainty in reservoir characterization, making traditional history matching both time-consuming and non-unique. These geological complexities, combined with data constraints, render conventional standalone simulation approaches inadequate for rapid, reliable waterflood optimization. Consequently, a hybrid framework that synergistically integrates physics-based modeling with machine learning offers a promising pathway to overcome these limitations, enabling faster scenario screening, improved parameter quantification, and more robust decision-making in this challenging environment.

**Fig. 1:**
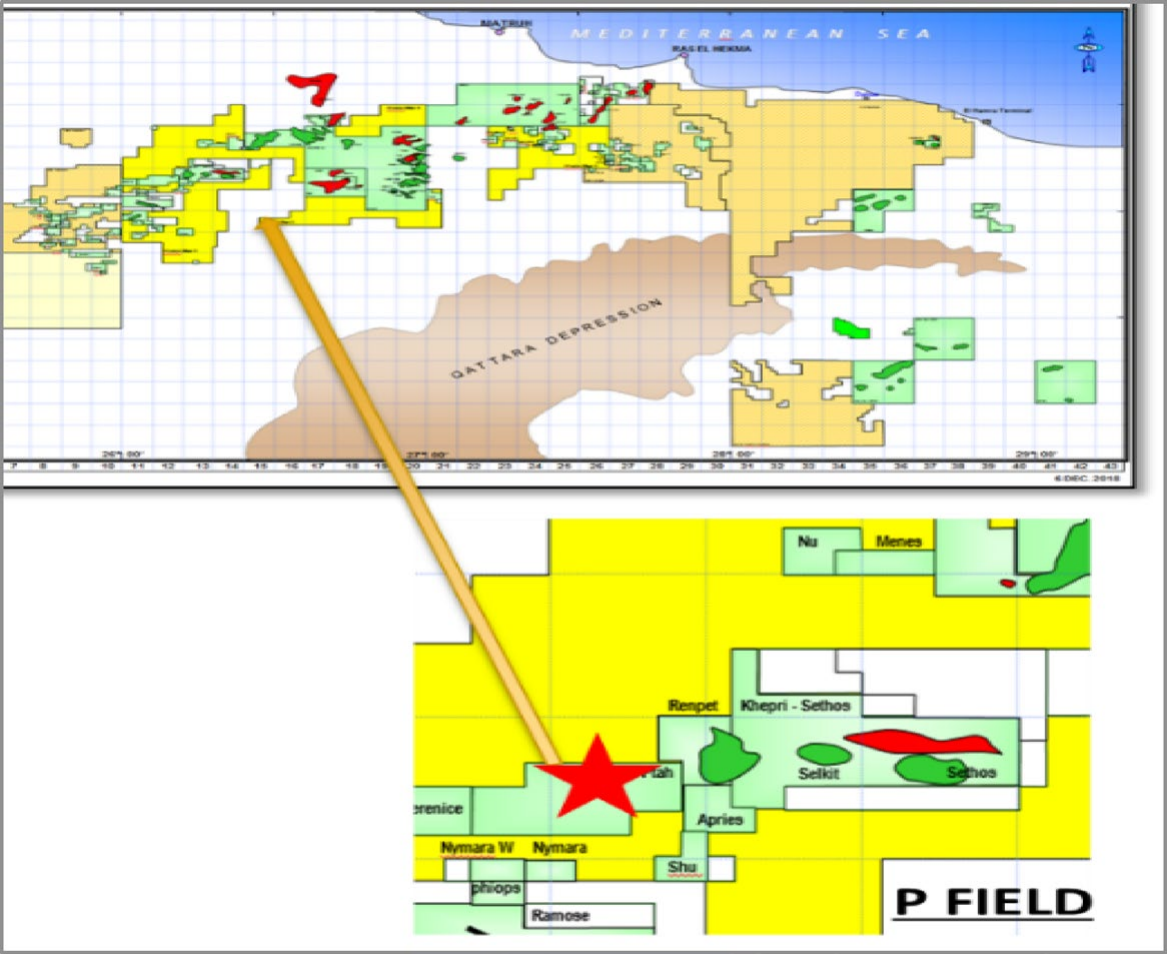
The P-Oil Field’s location map.

Despite these advances, a critical gap remains in the practical application of hybrid AI-numerical methods for routine decision-making in complex, data-constrained environments like Egypt’s Western Desert. Many existing models are either too computationally intensive for rapid screening or lack the interpretability required for engineers to trust and act upon their recommendations. Therefore, this study addresses this gap by developing a streamlined, hybrid framework that combines traditional reservoir simulation with machine learning-driven analytics. Focusing on the Bahariya Formation in Egypt’s Western Desert (Fig. 1), this work aims to evaluate multiple injection patterns, optimize waterflood design, and identify the controlling geological and operational parameters influencing oil recovery efficiency, ultimately providing field engineers with an actionable framework for enhanced reservoir management.

### The evolving role of AI/ML in modern reservoir development and management

The future of reservoir development and management is being actively reshaped by the digital transformation of the oil and gas industry, with artificial intelligence and machine learning serving as key enablers of enhanced efficiency and decision making^21,22^. It is now well established that major international and national oil companies (e.g., BP, ExxonMobil, Shell, Aramco, ADNOC) routinely employ advanced automated workflows for various subsurface tasks^21,22^. Consequently, the conversation has shifted from introducing AI/ML as an emerging novelty to critically examining how these tools can be further refined, integrated, and automated to deliver continuous improvements in business performance.

A central area of development is the application of advanced data analytics to optimize field development planning and reservoir management. AI/ML techniques are now applied across a broad spectrum of activities beyond secondary recovery, including:

- **Field Development Planning (FDP)**: Optimizing well placement, drilling schedules, and facility sizing under geological uncertainty^21,22^.
- **Production Forecasting**: Creating robust proxy models for rapid scenario analysis, as demonstrated in recent studies on unconventional reservoir performance^21,22^.
- **Enhanced Oil Recovery (EOR) Screening**: Rapidly evaluating the suitability of various EOR methods (e.g., chemical flooding, gas injection) based on reservoir characteristics^21,22^.
- **Automated History Matching**: Significantly reducing the time required to calibrate reservoir simulation models against production data^21,22^.

Technological advancements in downhole sensors and real-time monitoring systems are providing the granular surveillance data that feed into these data-driven workflows, enabling enhanced dynamic model calibration and adaptive control^21,22^.

In parallel with hydrocarbon recovery optimization, the industry is witnessing a distinct evolution in AI applications for carbon capture, utilization, and storage (CCUS/CCS). While sharing the common thread of subsurface modeling, CCUS workflows focus on different technical objectives, such as plume migration forecasting, storage capacity estimation, and long-term containment assurance. These applications utilize similar machine learning tools but address fundamentally different physical processes and risk profiles compared to production optimization^21,22^. The present study focuses specifically on enhancing AI-driven waterflooding for oil recovery, though the methodological framework could potentially be adapted for CCUS-related forecasting in future work.

The digitization trends discussed here, particularly the adoption of digital twins and automated reservoir management systems, are accelerating optimization and reducing operational costs. These developments collectively indicate a paradigm shift toward intelligent, adaptive reservoir management systems capable of maximizing resource recovery while addressing economic constraints^21,22^. The hybrid AI numerical approach developed in this study contributes to this evolution by demonstrating how such methods can deliver practical value for waterflood optimization in complex reservoirs today, while establishing a foundation for integrating more advanced capabilities as the technology matures.

### Machine learning-driven proxy modeling for waterflooding optimization in complex reservoirs

Machine learning has emerged as a valuable complementary tool in reservoir engineering, offering data-driven capabilities for waterflood optimization in heterogeneous formations^23,24^. ML techniques, including neural networks, decision trees, and ensemble methods, can capture complex subsurface patterns that traditional modeling approaches may overlook^25^. Applications of ML in waterflooding include reservoir property prediction, production forecasting, and dynamic optimization of injection strategies^26,27^.

A particularly impactful application is the development of ML-based proxy models, simplified representations that emulate the behavior of complex numerical simulators with significantly reduced computational cost. These proxy models enable rapid scenario testing, uncertainty quantification, and probabilistic forecasting, transforming how reservoir engineers approach field development planning.

The literature demonstrates growing sophistication in ML-proxy applications for enhanced oil recovery. Foundational work established best practices for proxy modeling in reservoir simulation, demonstrating that response surface methodologies can effectively substitute for full physics models in uncertainty quantification studies^28^. Subsequent research advanced these concepts by bridging deterministic and probabilistic approaches through advanced proxy-based methods^29^. These foundational studies established the theoretical framework upon which subsequent applications have been built.

For gas injection processes specifically, ML-based optimization for injection rate control demonstrated that reduced order models can maintain accuracy while dramatically decreasing computational requirements^30^. Proxy models for miscible CO₂-WAG injection in heterogeneous clastic reservoirs achieved robust predictions of recovery performance under various operational scenarios using the Box-Behnken design^31^. These concepts were extended to partially depleted oil reservoirs, optimizing CO₂-EOR processes using response surface methodologies that accounted for geological uncertainties^32^.

Hybrid genetic algorithm fuzzy logic systems have been applied to improve oil recovery in sandstone reservoirs, illustrating the value of evolutionary algorithms for optimization under uncertainty^33^. This approach demonstrated that combining fuzzy logic’s ability to handle imprecise information with genetic algorithms’ global search capabilities could effectively identify optimal operating conditions. Proxy modeling applications have also been expanded to thermal recovery methods, developing time-dependent models for SAGD processes that captured the dynamic nature of steam evolution^34^.

More recent advances have focused on integrating experimental design with sophisticated machine learning algorithms. Studies integrating experimental design with four machine learning algorithms, Quadratic Equation, Fuzzy Logic-Genetic Algorithm, Multivariate Additive Regression Splines (MARS), and Generalized Boosted Modeling (GBM) demonstrated that advanced machine learning can effectively replace complex compositional simulations while maintaining prediction accuracy, with GBM achieving exceptional accuracy (adjusted R² of 0.9973)^35^. Concurrently, combining Particle Swarm Optimization (PSO) with polynomial and nonparametric regressions for cyclic CO₂ flooding optimization achieved significant incremental oil production increases through optimization of operational parameters, including injection, soaking, and production durations^16,36^. Comparative analysis revealed that polynomial regression provided the least prediction error among the tested approaches.

Recent experimental validation provided critical laboratory confirmation of CO₂-assisted gravity drainage processes, bridging the gap between numerical simulation and physical reality^37^. This work demonstrated that properly calibrated proxy models could accurately predict recovery mechanisms observed in controlled laboratory settings. Additional studies have further expanded the application domain of ML proxy approaches across diverse reservoir types and recovery processes.

Collectively, these studies establish that hybrid ML-numerical workflows can effectively balance computational efficiency with predictive accuracy, a principle directly applicable to waterflood optimization in complex clastic reservoirs.

Despite these advances, reservoir heterogeneity and geological uncertainty remain fundamental challenges in waterflood design, particularly in complex clastic formations^26,27^. The Bahariya Formation exemplifies these difficulties, with pronounced permeability contrasts, discontinuous sand bodies, and limited well control creating substantial uncertainty in flow behavior prediction^26,27^. These complexities can result in uneven displacement fronts, bypassed oil, and early water breakthrough, reducing the predictive accuracy of conventional numerical simulations and hindering effective optimization^26,27^. ML-based seismic interpretation techniques have been shown to detect subtle stratigraphic and structural features, while automated well log analysis enhances lithofacies classification and petrophysical evaluation^38,39^.

This study directly addresses these challenges by developing a hybrid ML-proxy framework tailored to the Bahariya Formation’s specific geological characteristics. Unlike generic ML applications, our approach: (1) integrates formation-specific parameter distributions derived from field data, (2) quantifies pattern-dependent sensitivity through permutation importance analysis, (3) generates interpretable regression equations that provide engineering insights beyond black box predictions, and (4) validates predictions against numerical simulation benchmarks across three flooding patterns. By systematically combining high-fidelity numerical simulation with advanced ML algorithms, the workflow enables rapid evaluation of multiple injection scenarios while maintaining physical consistency, a critical requirement for decision-making in data-constrained environments like Egypt’s Western Desert.

### Integrating reservoir simulation and AI techniques for improved waterflooding optimization

Waterflooding optimization in mature fields aims to maximize oil recovery through controlled injection, traditionally relying on numerical reservoir simulation. While these physics-based models are invaluable, their effectiveness is constrained by incomplete datasets, simplifying assumptions, and computational complexity^13^. Integrating artificial intelligence, particularly machine learning, addresses these limitations by leveraging historical data to model nonlinear relationships between reservoir characteristics and waterflooding outcomes^13^.

A significant advantage of this integration lies in improved predictive fidelity. ML algorithms can model complex multiphase flow dynamics more efficiently than purely physics-based simulators, enabling real-time optimization of well placement and injection allocation^13^. However, successful integration requires high-quality, well-curated datasets to avoid overfitting, and models must be continuously validated against surveillance data^38,39^.

**Fig. 2:**
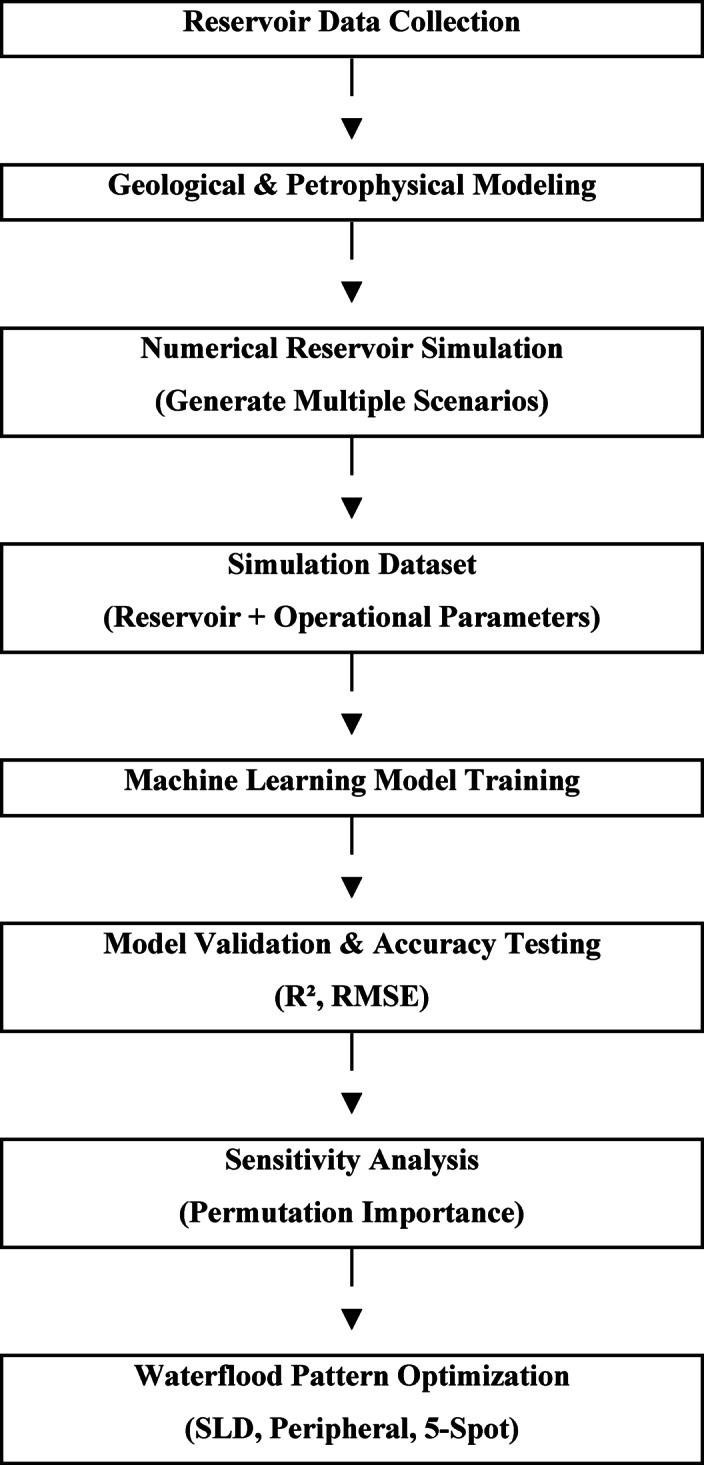
Overall hybrid workflow.

Figure 2 illustrates the overall hybrid workflow used in this study. The process begins with reservoir model construction and simulation-based scenario generation, followed by machine learning model training, validation, and performance evaluation. Finally, the trained models are applied to analyze parameter sensitivity and optimize waterflood design. This integrated approach allows efficient evaluation of multiple injection patterns while maintaining the physical realism provided by reservoir simulation.

Key benefits of this integrated approach include:

1. More Accurate Reservoir Forecasting: AI algorithms improve simulator calibration by identifying relationships between uncertain parameters (permeability distribution, fluid properties) and field performance.
2. Dynamic Production Optimization: ML-driven optimization enables continuous updates to injection rates, improving sweep efficiency and reducing water cut.
3. Cost and Risk Reduction: Predictive analytics minimize unnecessary drilling and optimize completion strategies.

This synergistic approach is crucial for mature oilfields with complex geologies, such as the Bahariya Reservoir in Egypt’s Western Desert, where integration enables data-driven, physics-constrained decision-making.

### Case studies of AI–simulation integration in waterflooding

- Hybrid Physics-Embedded Machine Learning for Large-Scale Waterflood Optimization^40^.

A recent field case study in Montana demonstrated the application of a physics-embedded machine learning workflow for optimizing waterflood operations. The approach combined conventional reservoir simulation with machine learning models trained on historical production and injection data to generate rapid predictive forecasts. This hybrid framework enabled large-scale scenario testing and Pareto-front optimization across thousands of operational configurations, allowing engineers to evaluate injection strategies in minutes rather than hours or days. The workflow significantly improved decision-making efficiency and supported the implementation of optimized water injection strategies in the field. This study provides a relevant precedent for the present work, where numerical reservoir simulation is similarly used to generate training datasets for machine learning models that rapidly evaluate multiple waterflooding scenarios.

- Machine Learning Enabled Sensitivity Analysis of Low-Salinity Waterflood with CO₂ Injection^41^.

Another recent study applied advanced machine learning algorithms, including CatBoost and LightGBM, to evaluate the performance of low salinity waterflooding combined with immiscible CO₂ injection in a sandstone reservoir. The ML models were trained on extensive reservoir datasets to perform sensitivity analysis and predict recovery outcomes under varying operational conditions. The results demonstrated that machine learning techniques can effectively identify nonlinear relationships between reservoir parameters and recovery performance, providing valuable insights for optimizing enhanced oil recovery processes. This approach is consistent with the methodology adopted in the present study, where machine learning models are used to perform sensitivity analysis and identify the key geological and operational parameters controlling recovery efficiency in different waterflood patterns.

## Materials and methods

**Fig. 3:**
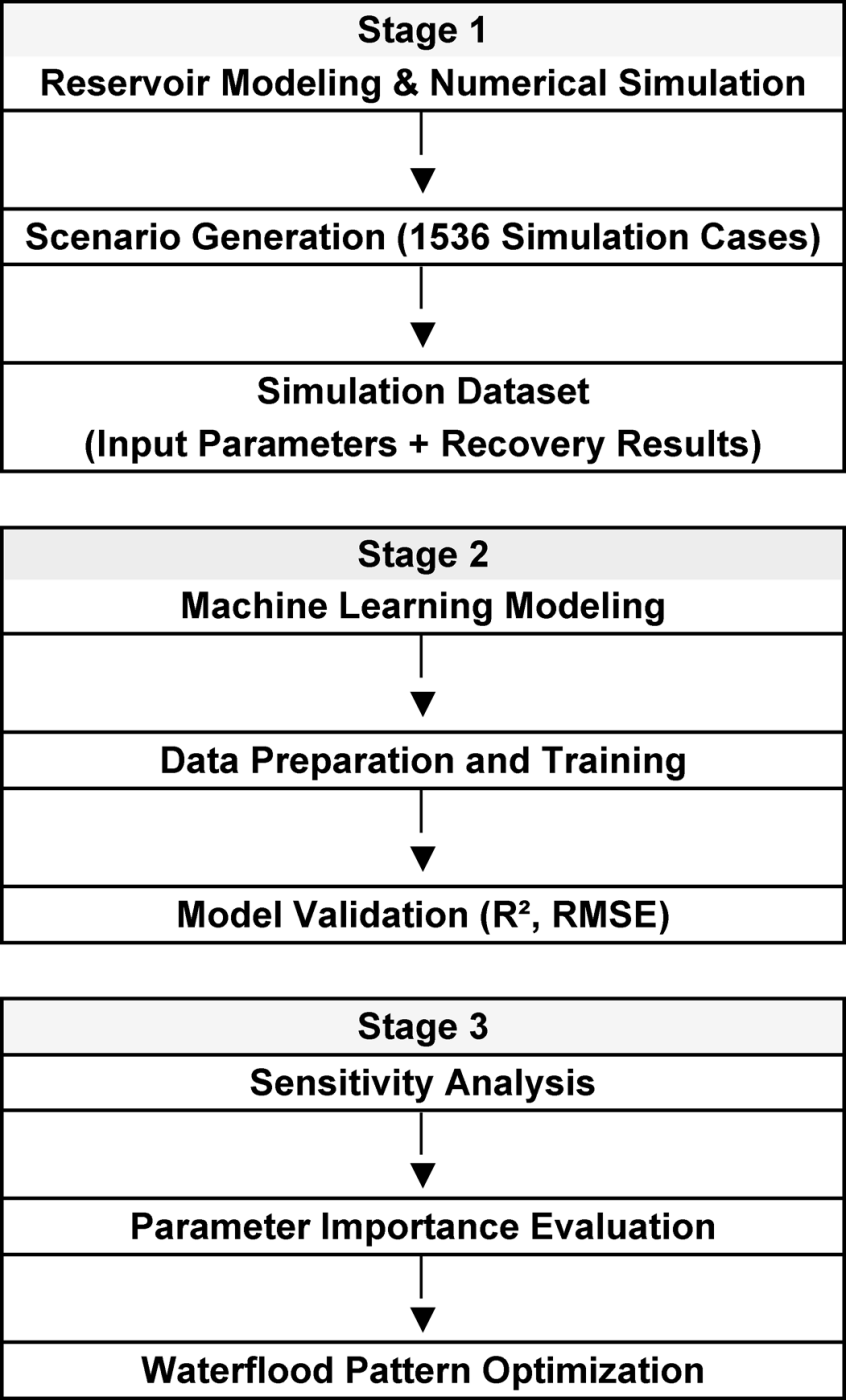
Overall hybrid workflow stages.

This study employs a hybrid workflow that integrates numerical reservoir simulation with machine learning based analysis to evaluate and optimize waterflooding strategies. The methodology consists of three main stages: (1) reservoir simulation and scenario generation, (2) machine learning model development, and (3) sensitivity analysis and performance evaluation. The overall workflow is summarized in Fig. 3.

### Study area and geological framework

**Fig. 4:**
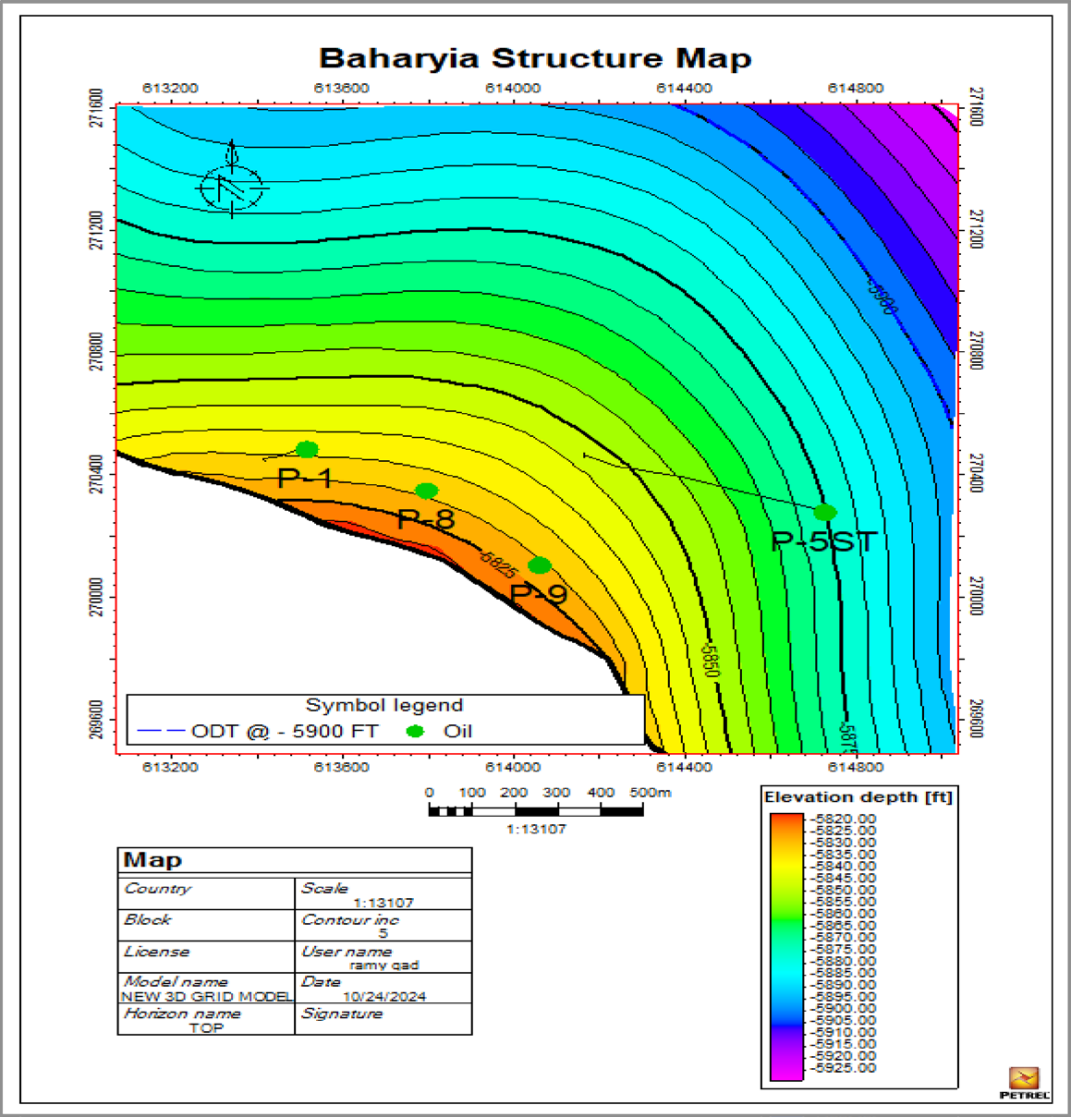
Baharyia Reservoir Structure Contour Map.

The Bahariya Formation in the Western Desert of Egypt is a Cenomanian-age clastic reservoir characterized by significant lithological variability and heterogeneous sandstone bodies (Fig. 4). The formation is typically divided into two main stratigraphic intervals:

- Lower Bahariya: Predominantly fluvial to shallow marine sandstones with relatively higher reservoir quality.
- Upper Bahariya: More argillaceous with interbedded shales and siltstones, reflecting a deltaic depositional environment.

The depositional setting results in complex sand body connectivity and lateral reservoir heterogeneity, which can significantly influence fluid flow behavior during waterflooding operations. Petrophysical evaluation from open hole log analysis indicates that the Bahariya reservoir contains hydrocarbon-bearing sandstone intervals with the following average properties:

- Net-to-Gross (NTG): 0.518, indicating that approximately half of the reservoir interval consists of productive sandstone layers.
- Porosity (PHI): 0.22, representing moderate pore volume capable of storing hydrocarbons.
- Initial Water Saturation (Swi): 0.27, suggesting a relatively high oil saturation within the reservoir pore space.
- Permeability (K): 148 mD, reflecting moderate permeability that supports fluid flow and water injection.
- Oil Down To (ODT): −5900 ft SSTVD.

These values indicate a moderate-quality sandstone reservoir with sufficient permeability and porosity to support waterflood development, although heterogeneity may influence sweep efficiency. The reservoir contains medium-gravity crude oil with a moderate gas–oil ratio (GOR), typical of clastic reservoirs in the Western Desert.

### Stage 1: Numerical reservoir simulation and scenario generation

A calibrated 3D black-oil reservoir simulation model was developed to evaluate waterflood performance under different injection patterns.

The geological model was constructed using Petrel™ (Schlumberger) for structural modeling, grid generation, and petrophysical property distribution, including porosity, permeability, and net to gross ratio. Dynamic flow simulation was performed using a black-oil reservoir simulator to reproduce historical production behavior and generate predictive development scenarios.

The simulation model was history matched using production and pressure data. Key adjustable parameters included relative permeability endpoints and horizontal permeability. The quality of the history match was evaluated using the Normalized Root Mean Square Error (NRMSE), which was maintained below 10%, indicating acceptable agreement between simulated and observed data.

To systematically explore reservoir and operational uncertainties, a Design of Experiments (DoE) approach was applied. Three waterflooding patterns were evaluated:

- Five-Spot pattern.
- Staggered Line Drive (SLD).
- Peripheral flooding.

Several key parameters were varied within realistic ranges to generate multiple simulation scenarios:

Injection parameters.

- Water injection rate (500–2000 bbl/day).

Geological parameters.

- Residual oil saturation (0.22–0.40).
- Water relative permeability (0.05–0.40).
- Initial water saturation (0.12–0.30).
- Horizontal permeability (150–500 mD).

Fluid properties.

- API gravity (31–38).

Operational constraints included bottom-hole pressure limits and an economic water cut limit of 95%. This process generated 1536 simulation scenarios, each representing a unique combination of reservoir and operational parameters. The resulting dataset included the input variables and the corresponding Ultimate Oil Recovery (UOR) obtained from the simulation results.

### Stage 2: Machine learning modeling

Machine learning models were developed to analyze the simulation dataset and identify the most influential parameters controlling recovery performance. The input variables consisted of geological and operational parameters obtained from the simulation design, while the target output variable was the simulated oil recovery efficiency.

The dataset was prepared using Python-based data processing tools. Simulation outputs and input parameters were merged using the simulation run identifier to create a unified dataset for model training and analysis.

To improve data quality, non-informative variables and redundant metadata columns were removed. Missing values were handled through complete case analysis, ensuring that only fully defined simulation scenarios were included in the modeling dataset.

Separate datasets were analyzed for each injection pattern (Five-Spot, Staggered Line Drive, and Peripheral flooding) to capture pattern-specific relationships between reservoir parameters and recovery performance.

#### Data collection and preprocessing

Input variables included geological and operational parameters such as permeability, residual oil saturation, injection rate, water saturation, and API gravity. The output variable represented the oil recovery efficiency derived from the simulation results.

Data preprocessing involved merging simulation outputs with the corresponding input parameters, removing redundant fields, and ensuring dataset consistency before machine learning analysis.

#### Exploratory data analysis (EDA)

An exploratory data analysis was performed to examine the statistical properties of the dataset and identify relationships between input parameters and recovery performance. Histograms were generated to visualize the distribution of key variables, while correlation analysis was used to evaluate potential relationships among parameters.

**Fig. 5:**
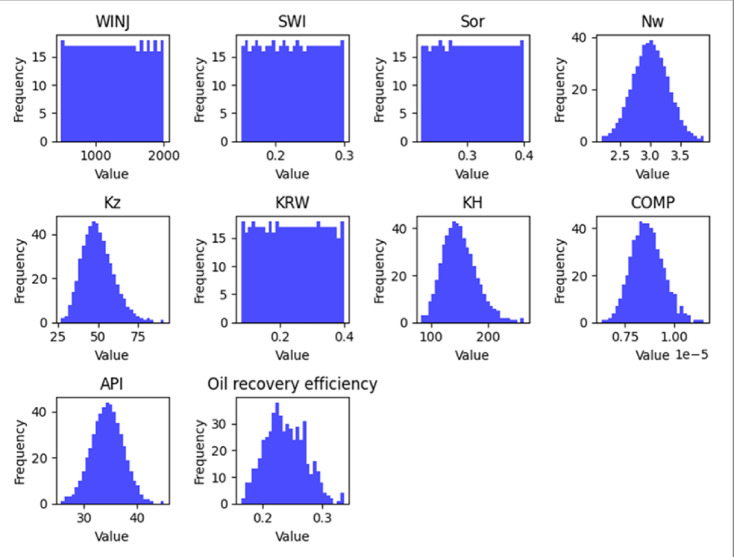
5-Spot Feature Histograms.

**Fig. 6:**
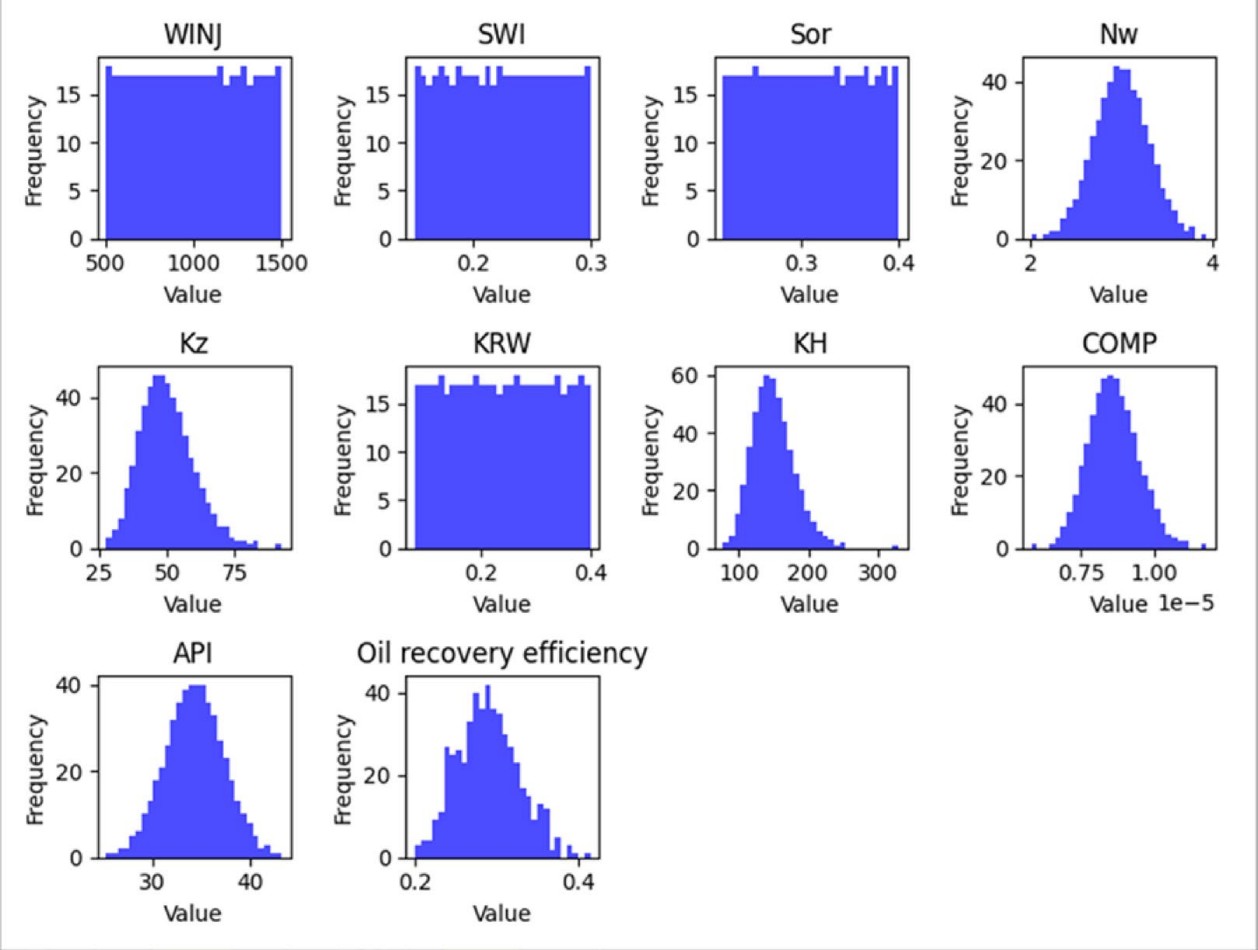
Peripheral Feature Histograms.

**Fig. 7:**
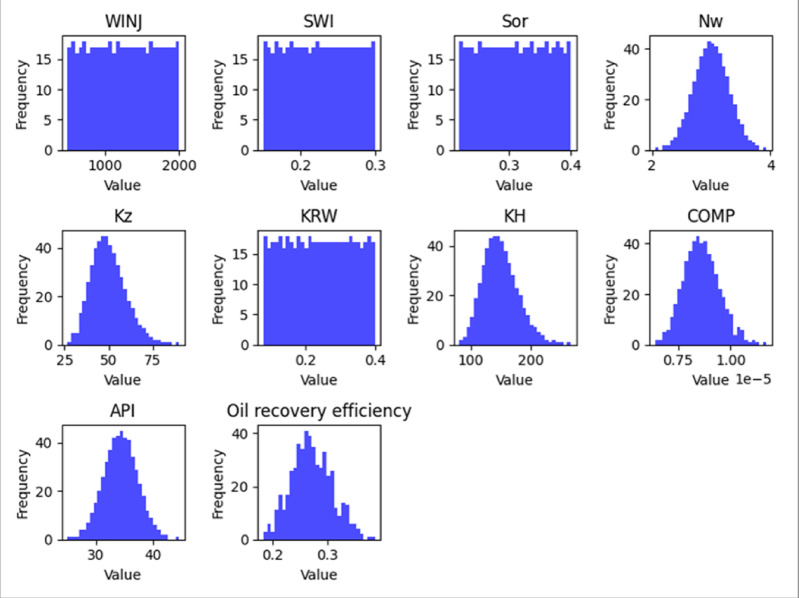
SLD Feature Histograms.

The EDA results, shown in Figs. 5, 6 and 7, provide an overview of parameter distributions and potential correlations that informed the subsequent machine learning modeling process.

**Fig. 8:**
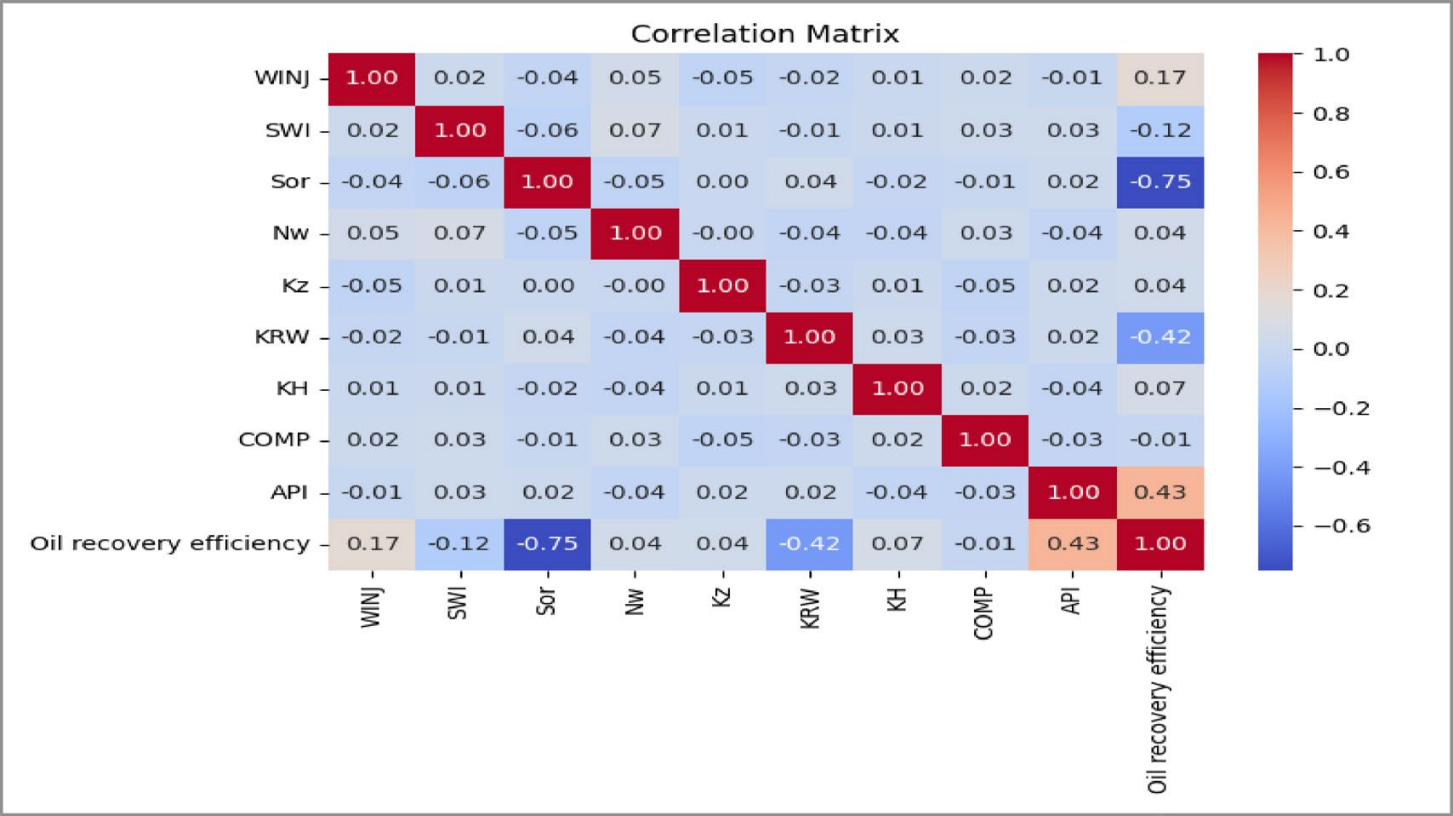
5-Spot Correlation Matrix.

**Fig. 9:**
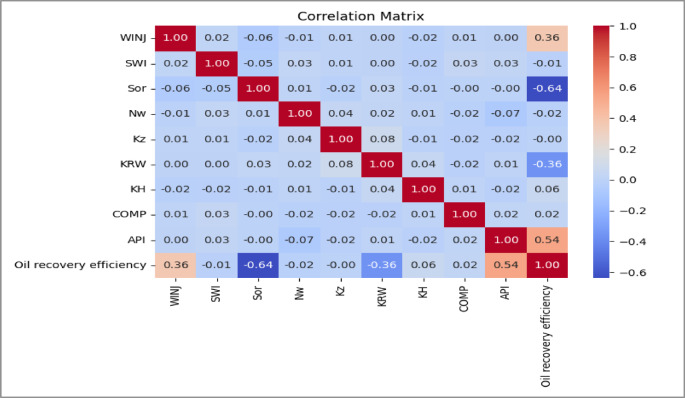
Peripheral Correlation Matrix.

**Fig. 10:**
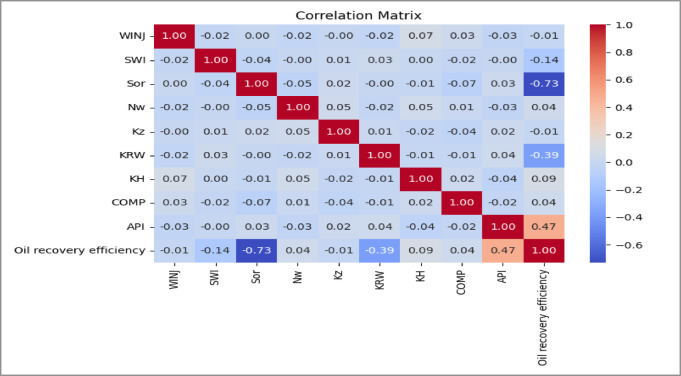
SLD Correlation Matrix.

Correlation Matrices as shown in Figs. 8, 9 and 10. Pairwise correlation matrices were computed and visualized as heatmaps to quantify linear relationships between all variables. This analysis was vital for detecting multicollinearity among input features, which can inflate model variance and complicate interpretation. For instance, a strong negative correlation was observed between residual oil saturation (S_or_) and recovery efficiency, a physically consistent and expected relationship that validates the dataset.

**Fig. 11:**
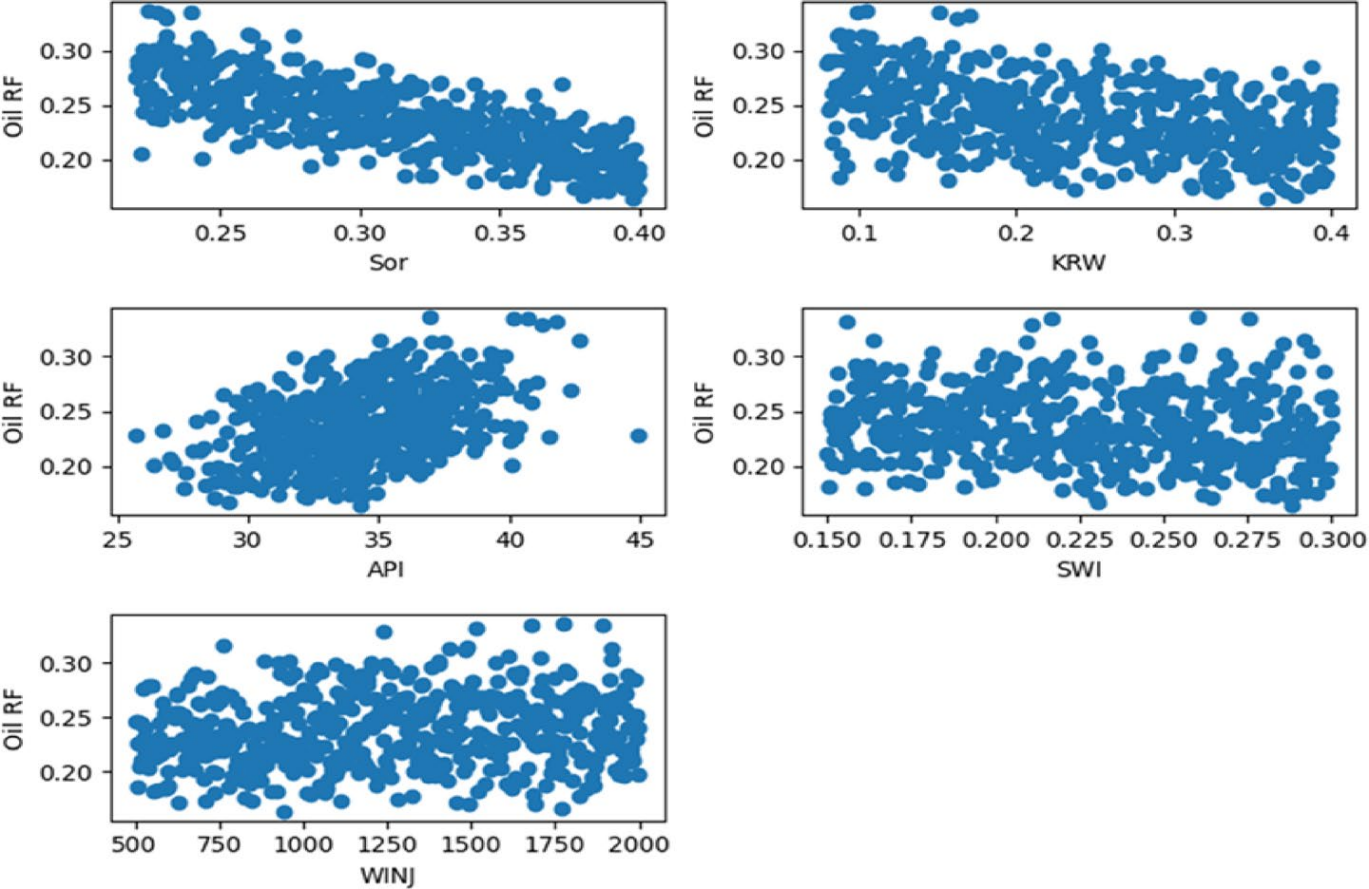
5-Spot Main Factors Scatter Plots.

**Fig. 12:**
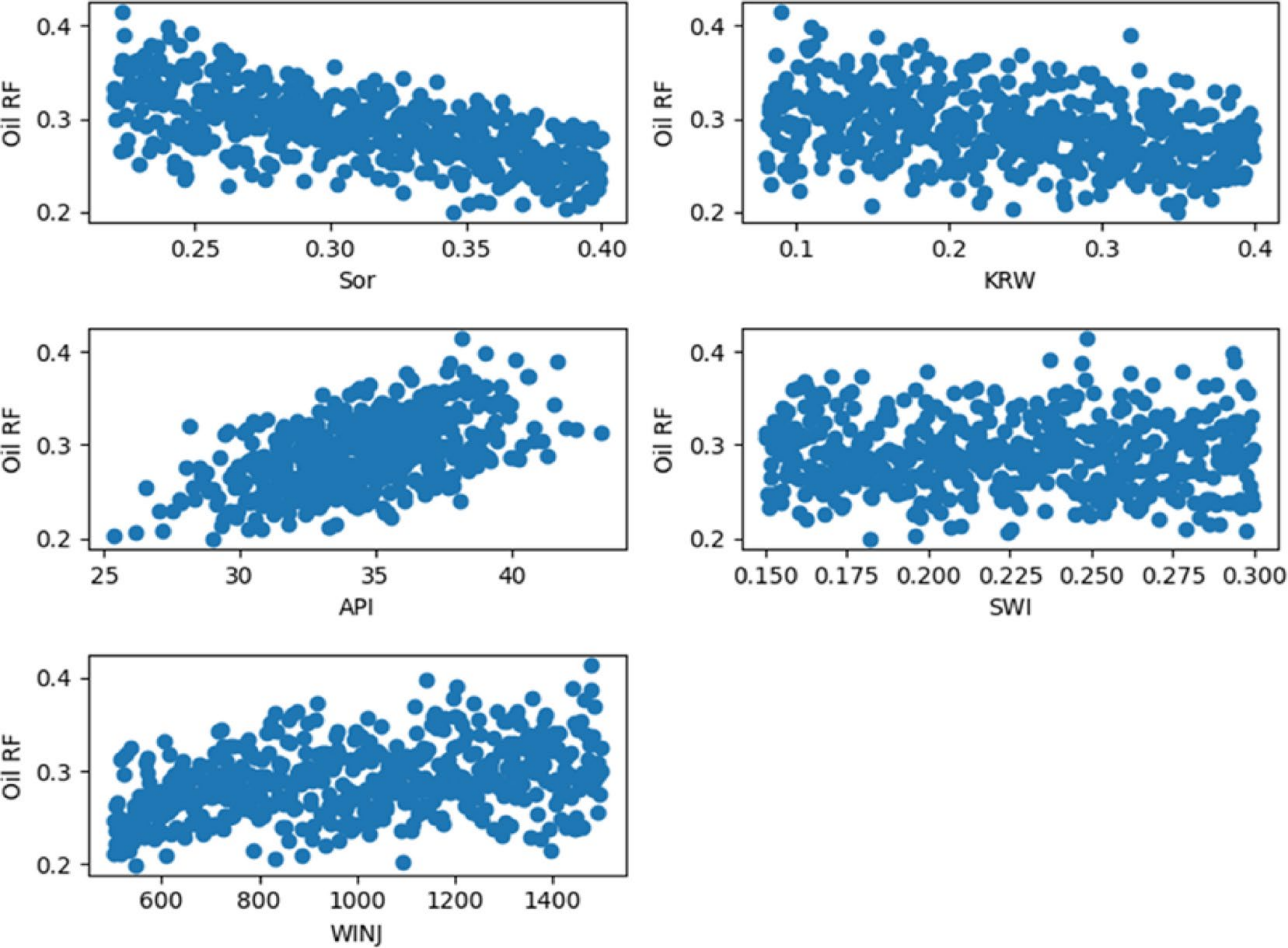
Peripheral Main Factors Scatter Plots.

**Fig. 13:**
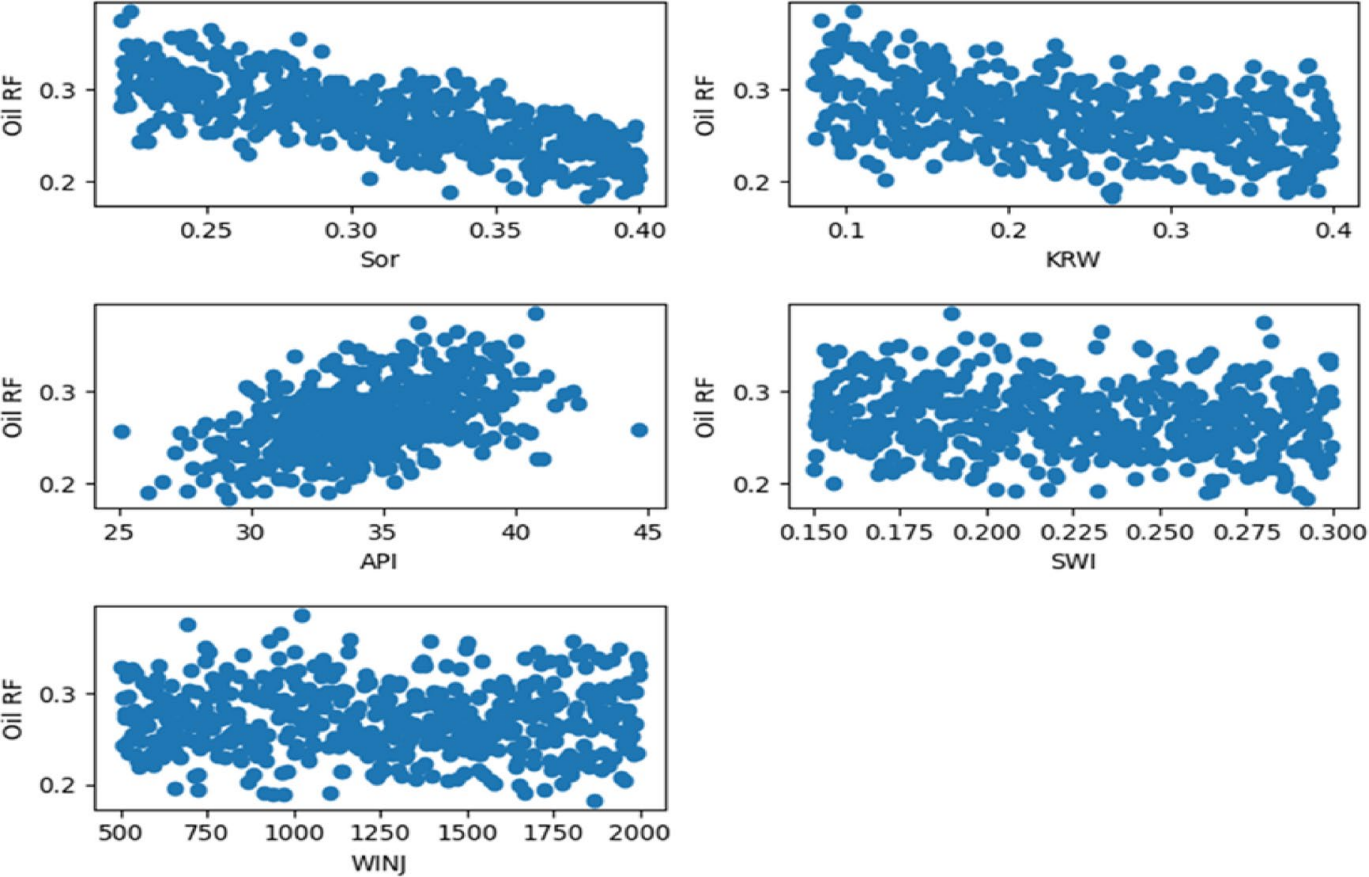
SLD Main Factors Scatter Plots.

Target-Oriented Scatter Plots as shown in Figs. 11, 12 and 13. To supplement the correlation analysis, scatter plots were generated to examine the specific functional relationships between the target variable (recovery efficiency) and three key features: residual oil saturation (S_or_), endpoint water relative permeability (K_rw_), and oil API gravity (API). These plots provide a granular view of the dependence of recovery performance on these critical parameters, revealing trends and non-linearities that are not fully captured by correlation coefficients alone.

### Model development

A linear regression model was developed to predict oil recovery efficiency using the preprocessed geological and operational parameters. The implementation was performed using the scikit-learn machine learning library in Python^42^. The dataset was divided into training and testing subsets using an 80:20 split to allow reliable model training and independent performance evaluation. To improve robustness and reduce sensitivity to a single data split, 5-fold cross-validation was applied during model training.

To rigorously validate our choice, we conducted a comprehensive sensitivity analysis evaluating model performance across five alternative train-test split ratios: 50:50, 60:40, 70:30, 80:20 (baseline), and 90:10. For each ratio, we performed 10 randomized iterations to account for sampling variability and computed mean R² and RMSE values across all three flooding patterns as shown in Table 1.

Based on this comprehensive sensitivity analysis, the 80:20 train test split was confirmed as the optimal choice for this study. This ratio:

**Table 1:** Model Performance Across Different Train Test Split Ratios (Mean ± Standard Deviation).

Split Ratio	Training Cases	Testing Cases	*R*² (Test Set)	RMSE (Test Set)
50:50	768	768	0.941 ± 0.008	0.0092 ± 0.0004
60:40	922	614	0.956 ± 0.006	0.0078 ± 0.0003
70:30	1075	461	0.967 ± 0.004	0.0065 ± 0.0002
80:20	**1229**	**307**	**0.973 ± 0.003**	**0.0057 ± 0.0002**
90:10	1382	154	0.978 ± 0.005	0.0054 ± 0.0003

- Maximizes the stability and reliability of performance metrics.
- Provides sufficient training data for robust model convergence.

**Fig. 14:**
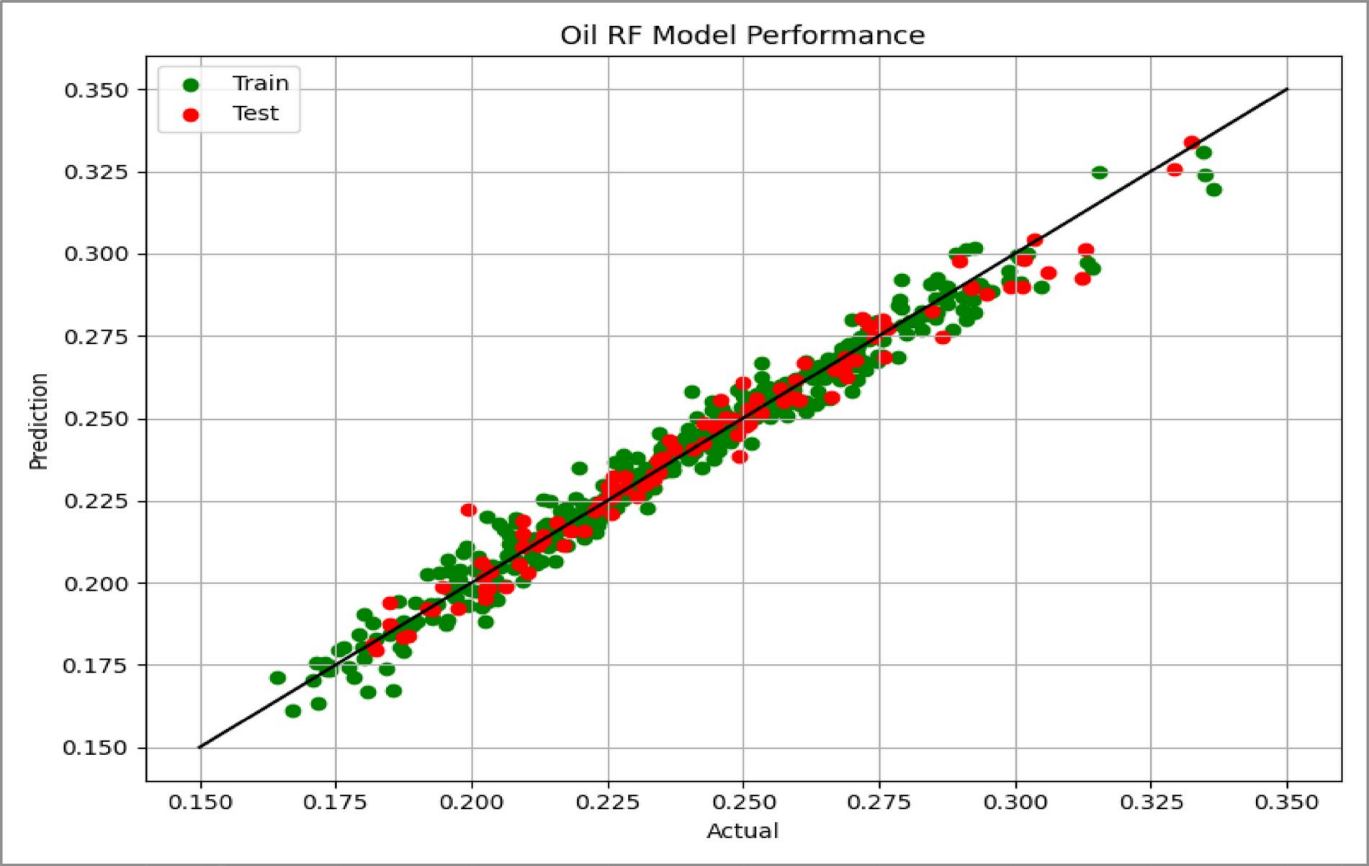
5-Spot Cross Plots Comparing Actual vs. Predicted Values.

**Fig. 15:**
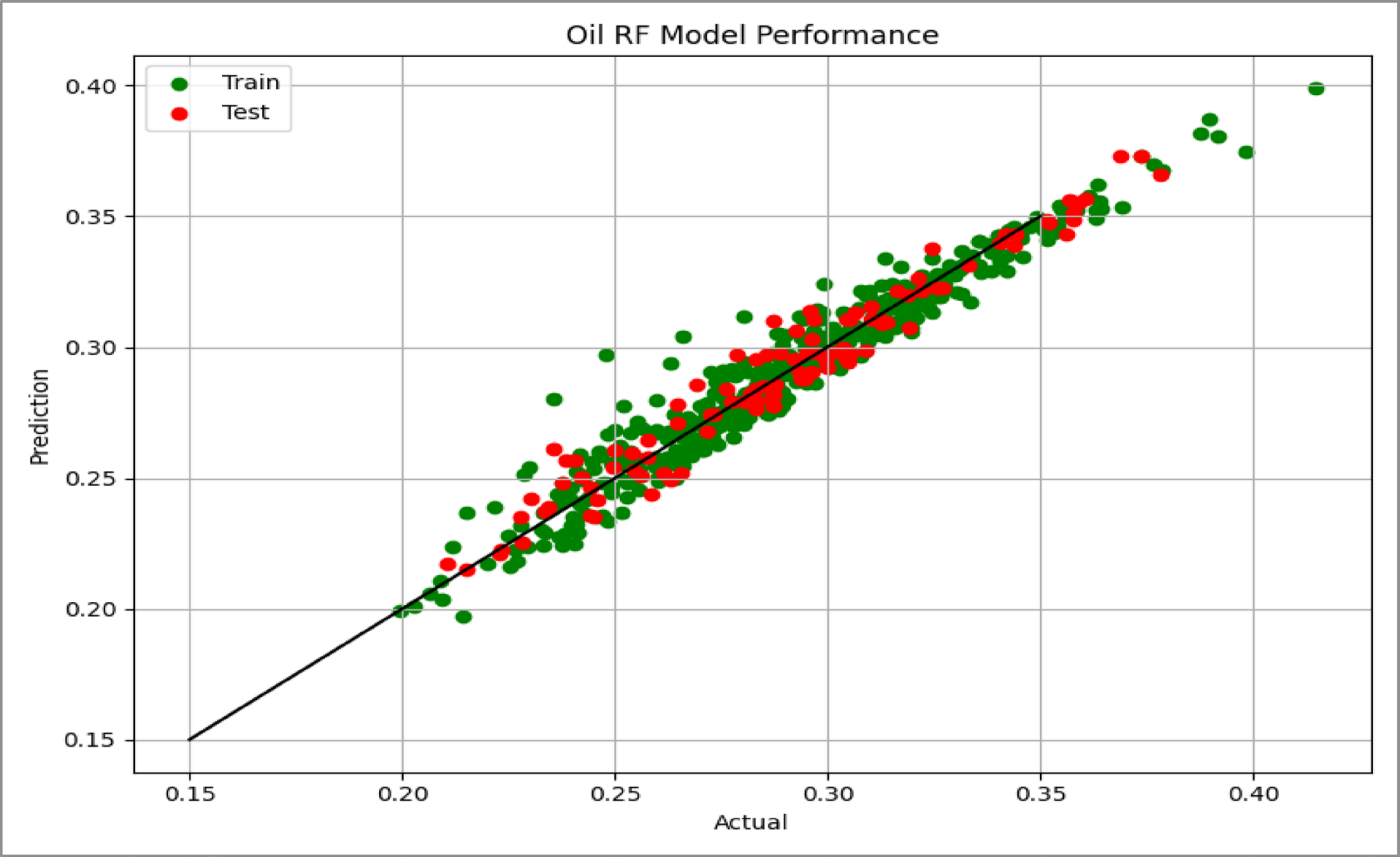
Peripheral Cross Plots Comparing Actual vs. Predicted Values.

**Fig. 16:**
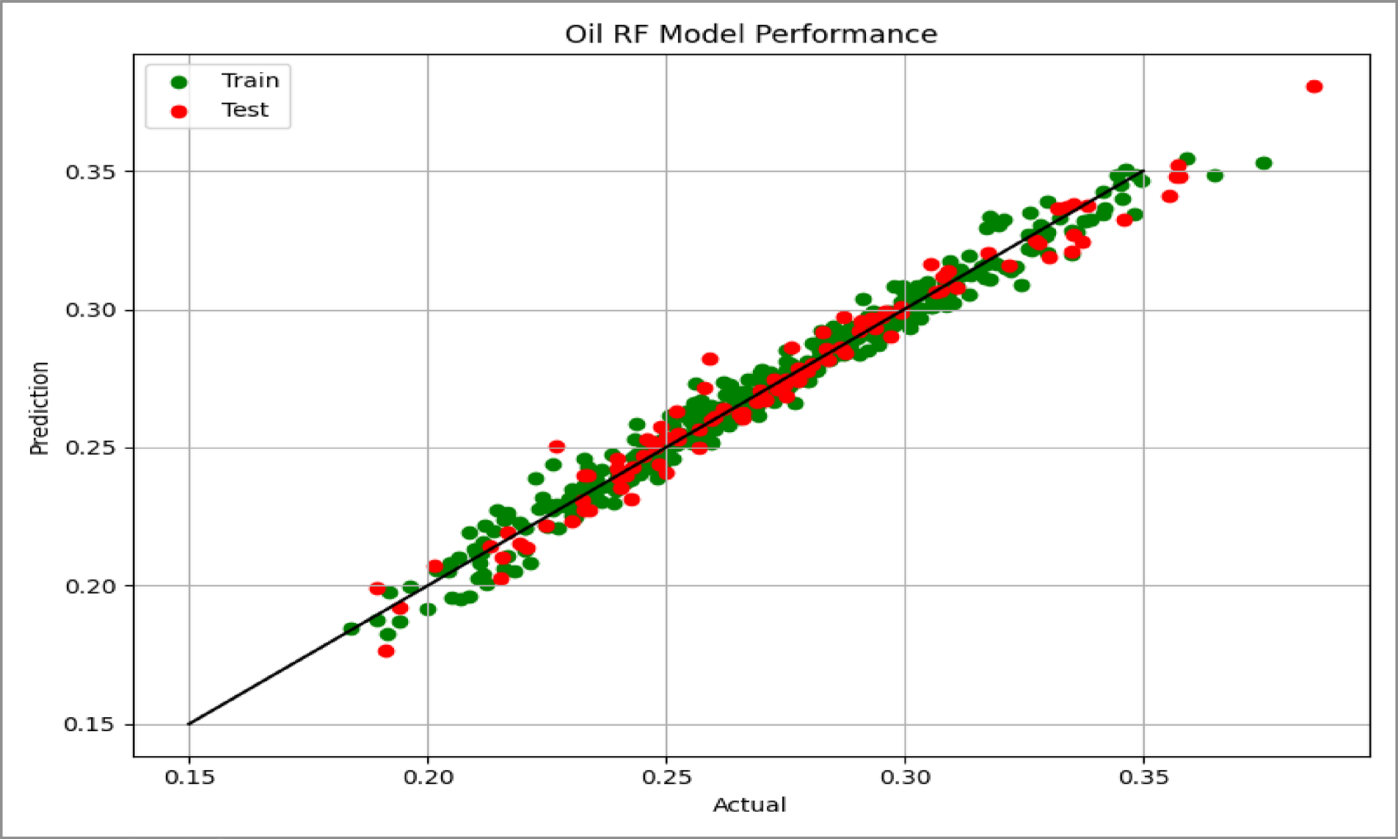
SLD Cross Plots Comparing Actual vs. Predicted Values.

Model performance was assessed using the coefficient of determination (R²) and Root Mean Squared Error (RMSE), which quantify the goodness of fit and the average prediction error, respectively. The predictive capability of the model was further evaluated using parity plots (actual vs. predicted values) for the test dataset, as shown in Figs. 14, 15 and 16, where points close to the unity line indicate good agreement between predicted and simulated recovery values.

## Results and discussion

### Model performance results

The performance of the linear regression models for the three injection patterns is summarized in Table 2. All models show strong predictive capability for estimating oil recovery efficiency.

**Table 2:** Performance metrics of linear regression models for each injection pattern case.

Case	Train *R*^2^	Test *R*^2^	CV *R*^2^	Train RMSE	TEST RMSE
5- Spot	0.972	0.973	0.970	0.0055	0.0057
SLD	0.976	0.972	0.972	0.0055	0.0068
Peripheral	0.939	0.953	0.934	0.0092	0.0085

All models achieved high coefficients of determination (R² > 0.93), indicating that the selected geological and operational parameters explain most of the variability in oil recovery efficiency. The 5-Spot and SLD models show the strongest performance, explaining approximately 97% of the recovery variation.

Prediction errors remain very small for all cases. The similarity between training and testing RMSE values suggests that the models are well calibrated and show no evidence of overfitting. In addition, the cross-validation scores closely match the training and testing results, confirming the stability of the models across different data partitions.

Overall, the results indicate that the regression models provide reliable predictions of recovery efficiency for different waterflood patterns.

### Analysis of feature importance

**Fig. 17:**
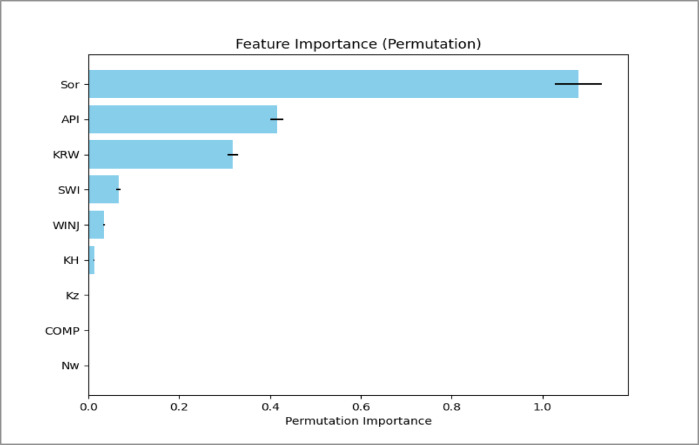
5-Spots Permutation Importance Analysis Plot.

**Fig. 18:**
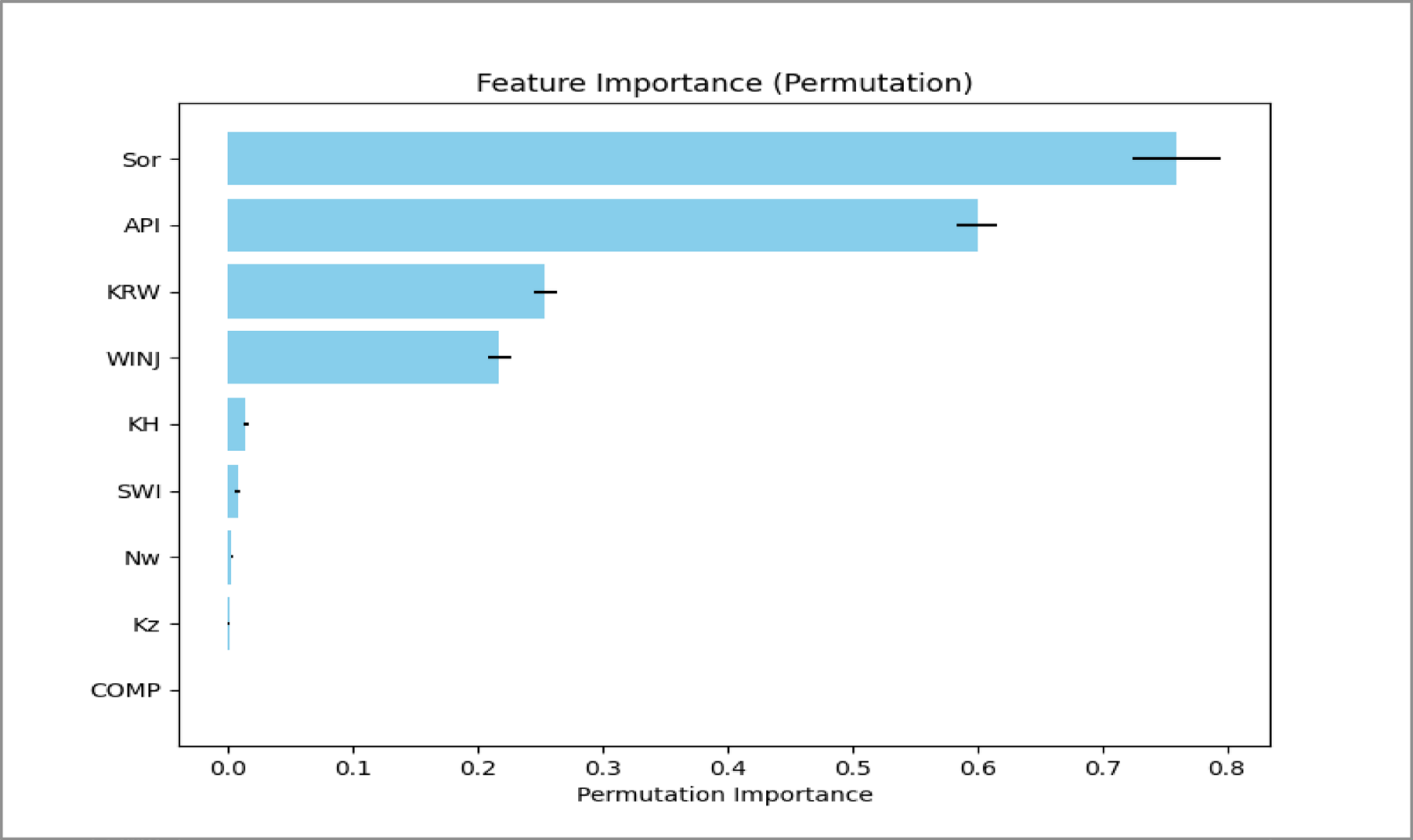
Peripheral Permutation Importance Analysis Plot.

**Fig. 19:**
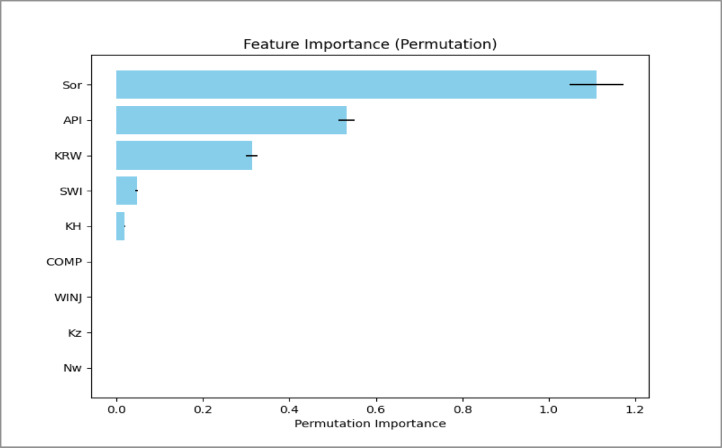
SLD Permutation Importance Analysis Plot.

Permutation importance analysis was used to identify the parameters that strongly influence oil recovery efficiency. The results are illustrated in Figs. 17, 18 and 19.

 Several consistent trends were observed across the three injection patterns.

- **Residual oil saturation (Sor)** is the most influential parameter. It shows a strong negative relationship with recovery efficiency. Higher Sor indicates that more oil remains trapped in the pore space, directly reducing the ultimate recovery factor.
- **API gravity** also has a positive influence on recovery. Lighter oils (higher API gravity) have lower viscosity and therefore flow more easily during water displacement, improving sweep efficiency.
- **Endpoint water relative permeability (Krw)** shows a negative impact on recovery. Higher water mobility can promote early water breakthrough and channeling in heterogeneous reservoirs, which reduces displacement efficiency.
- **The injection rate (WINJ)** plays a more important role in the peripheral flooding case than in the patterned floods. This suggests that recovery in peripheral injection schemes is more sensitive to injection pressure and vertical sweep, whereas the areal sweep in patterned floods is less dependent on injection rate within the studied range.

Regression Model Equations.

The Linear regression equations were extracted for each injection pattern to quantify the relationship between reservoir parameters and oil recovery factor. The full equations are provided in **Appendix A**, while the main trends are summarized here.

Across all three patterns, the coefficients show strong physical consistency with established reservoir engineering principles.

- **Residual oil saturation (Sor)** has the largest negative coefficient in all models, confirming its dominant influence on recovery. A reduction of Sor by 0.1 units can increase recovery by approximately 4–5% points, highlighting its importance for improved oil recovery strategies.
- **Water relative permeability (Krw)** also shows a negative effect, indicating that excessive water mobility reduces sweep efficiency and accelerates water breakthrough.
- **Initial water saturation (Swi)** negatively affects recovery because higher Swi reduces the initial volume of movable oil in the reservoir.
- **API gravity** consistently shows a positive contribution, confirming that lighter oil improves displacement efficiency and recovery performance.

These results demonstrate that the regression models capture the key physical relationships controlling waterflood performance.

### Field engineering implications

The results provide several practical insights for field development in heterogeneous sandstone reservoirs such as the Bahariya Formation. Engineers should prioritize reducing residual oil saturation and improving sweep efficiency, as these factors have the strongest influence on recovery. Controlling water mobility (Krw) and optimizing injection strategy, particularly in peripheral flooding schemes, can also significantly improve displacement efficiency. The developed regression models offer a rapid screening tool that allows engineers to evaluate waterflood scenarios and identify the most influential reservoir parameters before running computationally intensive simulation studies.

## Conclusion

This study successfully demonstrates the application of linear regression modeling for the prediction of oil recovery efficiency across different waterflooding patterns. The key conclusions are as follows:

1. Effective Predictive Modeling: Linear regression models proved to be highly effective, achieving excellent predictive accuracy with R² values exceeding 0.93 for all injection patterns (5-Spot, SLD, and Peripheral). This high performance confirms a strong linear relationship between the selected geological and operational parameters and recovery outcomes.
2. Dominant Predictive Features: Permutation importance analysis identified residual oil saturation (S_or_) as the most critical parameter negatively affecting recovery. This was followed by oil API gravity (API), which had a positive influence, and endpoint water relative permeability (K_rw_), which exhibited a negative impact likely due to water channeling.
3. Pattern-Dependent Dynamics: The importance of specific features was found to be influenced by the well configuration. Most notably, the water injection rate (WINJ) was a significantly more important predictor in the Peripheral flood case, underscoring how recovery mechanisms and their controlling parameters vary with injection pattern.

The findings highlight two main aspects of the study. First, the effectiveness of simple yet robust linear models demonstrates the value of machine learning as a complementary tool for reservoir engineering. These models serve as rapid, computationally efficient screening methods that provide early insights and help narrow the parameter space before conducting more detailed and resource-intensive numerical simulations. Second, the analysis revealed injection pattern-specific behaviors: the 5-Spot and SLD configurations exhibited slightly higher predictability due to their more uniform and confined sweep patterns, while the Peripheral flooding pattern showed a stronger dependence on injection rates, consistent with classical fluid flow dynamics in line-drive and edge-water injection systems.

Future work could extend this workflow to other reservoirs and formations in the Western Desert, and field pilot testing is recommended to validate the optimized waterflood strategies under real operating conditions.

## Data Availability

The data is available upon Request from the authors.

## Appendix A: Complete regression model equations

Five-Spot Model Equation:

$$\begin{aligned} Oil{\text{ }}RF{\text{ }} = & 0.00001*WINJ{\text{ }} - {\text{ }}0.14115*SWI{\text{ }} - {\text{ }}0.47716*Sor{\text{ }} + {\text{ }}0.00239*Nw{\text{ }} \\ & + {\text{ }}0.00010*Kz{\text{ }} - {\text{ }}0.14349*KRW{\text{ }} + {\text{ }}0.00009*KH \\ & - {\text{ }}916.93913*COMP{\text{ }} + {\text{ }}0.00511*API{\text{ }} + {\text{ }}0.24626~ \\ \end{aligned}$$

Peripheral Model Equation:

$$\begin{aligned} Oil{\text{ }}RF{\text{ }} = & 0.00004*WINJ{\text{ }} - {\text{ }}0.04769*SWI{\text{ }} - {\text{ }}0.44423*Sor{\text{ }} + {\text{ }}0.00470*Nw \\ & + 0.00005*Kz{\text{ }} - {\text{ }}0.14678*KRW{\text{ }} + {\text{ }}0.00011*KH{\text{ }} \\ & + 393.69227*COMP{\text{ }} + {\text{ }}0.00689*API{\text{ }} + {\text{ }}0.15669 \\ \end{aligned}$$

SLD Model Equation:

$$\begin{aligned} Oil{\text{ }}RF{\text{ }} = & - {\text{ }}0.000001*WINJ{\text{ }} - {\text{ }}0.13044*SWI{\text{ }} - {\text{ }}0.52920*Sor{\text{ }} + {\text{ }}0.00097*Nw \\ & - {\text{ }}0.00002*Kz{\text{ }} - {\text{ }}0.15887*KRW{\text{ }} + {\text{ }}0.00012*KH \\ & - {\text{ }}547.94109*COMP{\text{ }} + {\text{ }}0.00636*API{\text{ }} + {\text{ }}0.26962 \\ \end{aligned}$$

## Abbreviations

E&P: Exploration and Production
EDA: Exploratory Data Analysis
KPC: Khalda Petroleum Company
ML: Machine Learning
RMSE: Root Mean Squared Error
SLD: Staggered Line Drive
API: Oil API gravity (°API)
COMP: Rock compressibility (psi⁻¹)
K_H_: Permeability-thickness product (mD·ft)
K_rw_: Endpoint water relative permeability (fraction)
Kz: Vertical permeability (mD)
Nw: Number of injector wells
RF: Recovery efficiency, or recovery factor (fraction)
S_or_: Residual oil saturation (fraction)
S_wi_: Initial water saturation (fraction)
WINJ: Water injection rate (bbl/day)
R²: Coefficient of determination
X: Input variable/feature set
Y: Output/target variable
